# Epidemiologic Characterization of Human Papillomavirus Infection in Rural Chaozhou, Eastern Guangdong Province of China

**DOI:** 10.1371/journal.pone.0032149

**Published:** 2012-02-24

**Authors:** Qiang Chen, Long-Xu Xie, Zhi-Rong Qing, Lie-Jun Li, Zhao-Yun Luo, Min Lin, Shi-Ming Zhang, Wen-Zhou Chen, Bing-Zhong Lin, Qi-Li Lin, Hui Li, Wei-Pian Chen, Pei-Yao Zheng, Ling-Zhi Mao, Chan-Yu Chen, Chun Yang, Yong-Zhong Zhan, Xiang-Zhi Liu, Jia-Kun Zheng, Li-Ye Yang

**Affiliations:** 1 Central Lab, Chaozhou Central Hospital, Chaozhou, Guangdong Province, China; 2 Department of Radiobiology, School of Public Health, Jilin University, Changchun, China; 3 Chaozhou Hybribio Limited Corporation, Chaozhou, Guangdong Province, China; 4 Chaozhou Health Bureau, Chaozhou, Guangdong Province, China; 5 Raoping County People's Hospital, Raoping County, Guangdong Province, China; 6 Department of Gynecology and Obstetrics, Chaozhou People's Hospital, Chaozhou, Guangdong Province, China; 7 Department of Gynecology and Obstetrics, Chaozhou Gynecological and Pediatric Hospital, Chaozhou, Guangdong Province, China; 8 Chao'an County Gynecological and Pediatric Hospital, Chao'an County, Guangdong Province, China; 9 Fengxi People's Hospital, Chaozhou, Guangdong Province, China; 10 Department of Obstetrics and Gynecology, Guangdong Provincial Women and Children's Hospital, Guangzhou, China; 11 Department of Gynecology, Chaozhou Central Hospital, Chaozhou, Guangdong Province, China; Karolinska Institutet, Sweden

## Abstract

**Background:**

Human papilloma virus (HPV) infection was the main cause of cervical cancer. There were only a few reports and detailed data about epidemiological research of HPV infection in rural population of China.

**Materials and Methods:**

The cervical cells of rural Chaozhou women were collected, and multiplex real time PCR was firstly performed to detect high-risk HPV (HR-HPV) infection, which could detect 13 types of HR-HPV (types 16, 18, 31, 33, 35, 39, 45, 51, 52, 56, 58, 59, and 68). Then, HPV-positive samples were typed by HPV GenoArray test.

**Results:**

HR-HPV DNA was detected by multiplex real time-PCR in 3830 of 48559 cases (7.89%). There was a peak incidence in age of 55–60 years group, and a lower incidence in who lived in plain group compared with suburban, mountain and seashore group. 3380 cases of HPV positive sample were genotyped, 11.01% (372/3380) cases could not be classified, among the typed 3008 cases, 101 cases were identified without HR-HPV type infection, 2907 cases were infected with one HR-HPV type at least, the 6 most common HR-HPV types in descending order of infection, were type 52 (33.4%, 16 (20.95%), 58 (15.93%), 33 (9.94%), 68 (9.22%) and 18 (8.36%). The combined prevalence of HPV types 16 and 18 accounted for 28.52% of total infection. However, type 52 plus 58 presented 48.23% of total infection. 2209/2907 cases were infected with a single HPV type and 698/2907 cases were infected with multiple types, and multiple infection constituent ratio increased with age, with a peak incidence in age 55–60 years group.

**Conclusions:**

Our findings showed low prevalence of HPV vaccine types (16 and 18) and relatively high prevalence of HPV-52 and -58, support the hypothesis that the second-generation HPV vaccines including HPV-52 and -58 may offer higher protection for women in rural Guangdong Province.

## Introduction

Cervical cancer is the second most common cancer in women, about 200 000 deaths yearly are caused by cervical cancer in the world [1]. Therefore, the social consequences of this disease are still tremendous. Certain types of human papillomavirus (HPV) are now recognized as the main cause of cervical carcinoma [2]. At present, more than 100 HPV genotypes have been molecularly characterized [1]. According to previous report, HPV-16, -18, -31, -33, -35, -39, -45, -51, -52, -56, -58, -68, -69, -73, -59 and -82 were classified as “high-risk types”, associated with cervical cancer, while HPV-6, -11 -42, -43 and -44 were classified as “low-risk types” [2], [3]. The development of a prophylactic vaccine against these infections now appears to be the most promising way of controlling cervical neoplasia. There are two licensed HPV vaccines; Gardasil (HPV-6/11/16/18) and Cervarix (HPV-16/18), mainly against the two major cancer-causing types (HPV-16/18). These vaccines could also reduce the economic burden associated with HPV-related benign lesions, and these vaccines are safe, well tolerated and highly immunogenic [4]. Clinical trials have shown apparently type-restricted protective efficacy for genital lesions related to targeted types in women who were naive to the respective types at enrollment [4]–[8]. Since limited cross-protection could be offered between HPV types [9], heterogeneity in HPV type-specific distribution from different populations should be taken into account when predicting the effect of current prophylactic vaccines and forming the basis for the second-generation vaccines targeted to specific regions [10].

To the best of our knowledge, no epidemiologic data on HPV genotypes in general female population from rural area was reported from Guangdong Province, southern China, which highlights the need for timely population-based study in this region. Due to the accessible anatomic site for sample collection and the well-established sample collecting method for cervix, we investigated the cervical HPV infection in asymptomatic, general female population to understand the overall, type-specific, age-specific HPV prevalence and extent of multiple infections in rural Guangdong Province.

## Methods

### Study subjects

From December 2009 to September 2010, an epidemiologic screening for HPV infections was organized by Chaozhou municipal government, with the cooperation of gynaecological practitioners in 13 hospitals (Chaozhou Central Hospital, Chaozhou People's Hospital, Chaozhou Gynecological and Pediatric Hospital, Chao'an County Gynecological and Pediatric Hospital, Fengxi District Hospital, Raoping County People's Hospital, Raoping Huaqiao Hospital, Xinfeng People's Hospital, Qiaodong Hospital, Yixi Hospital, Chengxi Hospital, Fengxin Hospital and Raoping County Gynecological and Pediatric Hospital).

The population in rural Chaozhou eligible to this study included about 300000 women (aged from 35 to 60 years). The majority of eligible population refused to participate mainly because they did not have enough time or did not think they needed a gynecological examination in the absence of symptoms. A woman was eligible to be study subject if she: (a) was mentally and physically competent; (b) was aged between 35 and 60 years; (c) was a permanent resident of local area; (d) had no history of abnormal cytology or cervico-vaginal dysplasia; (e) had no history and associated symptoms of other HPV-related diseases; (f) had given birth at least once; (g) was not presently pregnant; (h) had not undergone a total uterus or cervix resection; (i) was willing to undergo HPV testing.

### Study design

**Figure 1:**
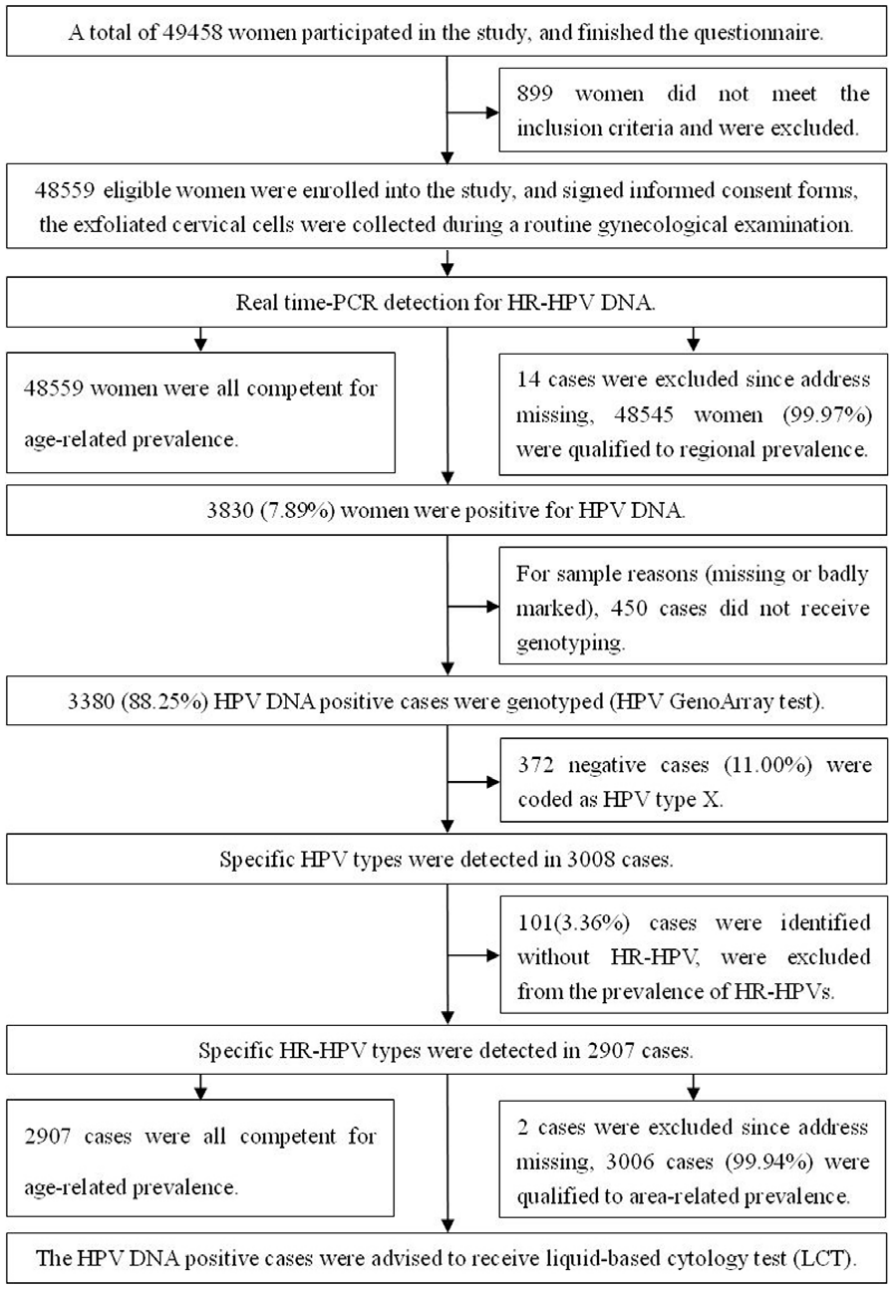
Enrollment and outcomes.

Eligible women were invited for a face-to-face interview, and a socio-demographic characteristics based questionnaire was designed to collect information on lifestyle, menstrual status, reproductive history, contraception and sexual behavior. Written informed consent was obtained by signature or thumbprint. After the interview, all women underwent a gynecological examination performed by gynaecological practitioners in their village clinics or in 13 above-mentioned hospitals, exfoliated cervical cells were collected for HPV DNA detection. Multiplex real time polymerase chain reaction (PCR) was firstly performed to detect high-risk HPV infection in 3 hospitals (Chaozhou People's Hospital, Raoping County People's Hospital, Chao'an County Gynecological and Pediatric Hospital). Then, HPV GenoArray test was used to detect the specific HPV subtypes with the same HR-HPV samples in Hybribio Limited Corporation, Chaozhou, China. HPV DNA positive cases for real time-PCR were advised to receive liquid-based cytology test (LCT), and histological diagnosis was performed if necessary. Most confirmed or highly suspicious high grade or invasive lesions were treated at the collaborating hospitals with loop excision, surgical conization, hysterectomy, or radiotherapy, according to local protocols. The screening and the following cytology test were provided at no cost to the participant. All studies were approved by Chaozhou Central Hospital Ethics Committee. The designed details were shown in Figure 1.

### Groups

**Figure 2:**
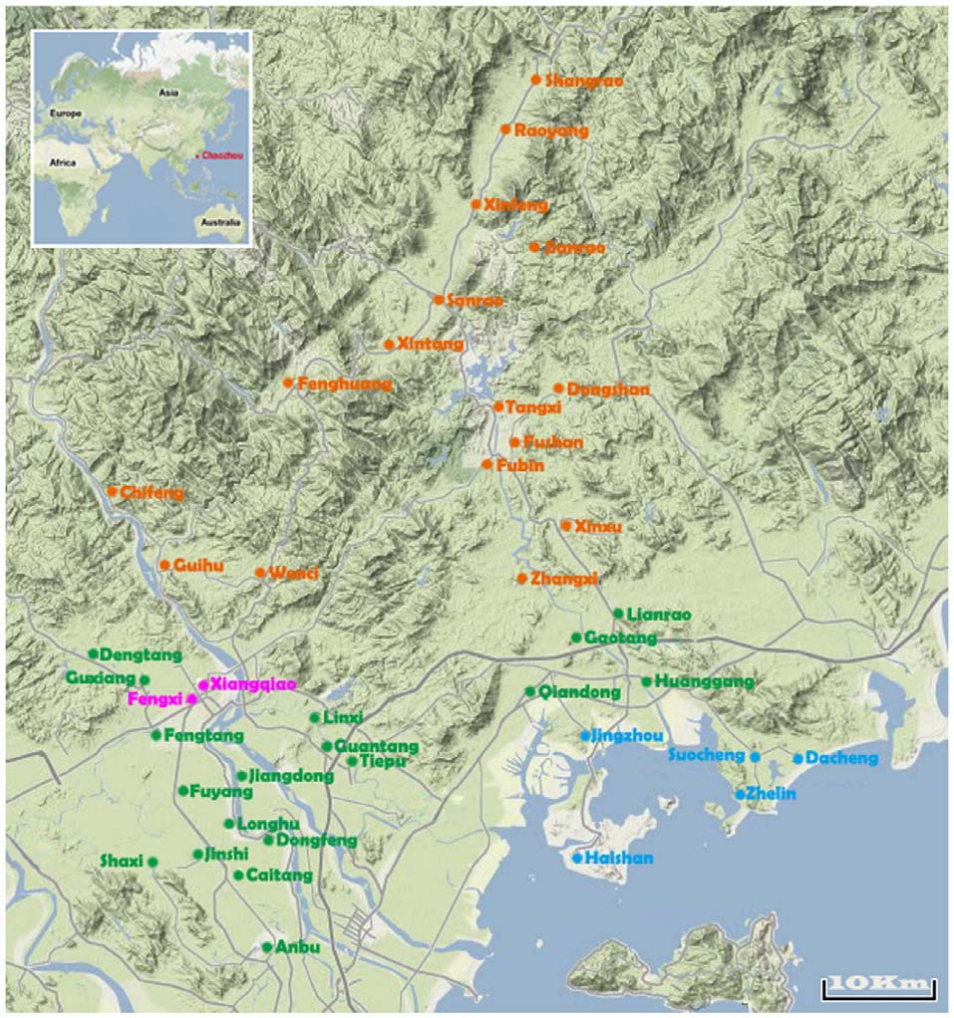
The topographic map of Chaozhou area. plain (green), suburban (pink), seashore (blue) and mountain (orange).

Chaozhou region is a place in easternmost Guangdong province of the People's Republic of China. It borders Shantou to the south, Jieyang to the southwest, Meizhou to the northwest, the province of Fujian to the east, and the South China Sea to the southeast. It is administered as a prefecture-level city with a jurisdiction area of 3,614 square kilometers and a total population of 2,600,000. Chaozhou's municipal region includes a city (Xiangqiao District and Fengxi District) and two county (Chao'an and Raoping County), with 840 natural villages (Figure 2).

For age prevalence, the eligible subjects were divided into 5 age groups with five-year interval, the actual age of each subject was counted with the formula: (interview data-birthday)/365.25 by Microsoft Excel software. For area prevalence, the eligible subjects were divided into 4 area groups and 41 clusters of natural villages, including suburban (2 clusters of villages), plain (18 clusters of villages), mountain (16 clusters of villages) and seashore (5 clusters of villages) (Figure 2) according to their living area.

### Cervical specimen collection and HPV DNA extraction

Samples of exfoliated cervical cells were collected using plastic cervical swabs. The sampler was inserted 1–1.5 cm into the endocervical canal and rotated 4–5 full turns in counter-clockwise direction. The tip containing cellular material was then placed into transport medium tube and stored at 4°C immediately. All specimens were coded without knowledge of the subjects. All swabs and store bottle with specimen transport medium were from Hybribio Biotechnology Limited Corp., Chaozhou, China. All specimens were finally sent to 3 clinical PCR laboratories (Chaozhou People's Hospital, Raoping County People's Hospital, Chao'an County Gynecological and Pediatric Hospital) for HPV analysis. The brush and supernatant were removed after the cells were centrifugated for 5 mins with relative centrifugal force 960 *g*. DNA was extracted from the sediments with alkaline lysis method Kits (Hybribio Biotechnology Limited Corp.), resuspended in 100 µL elution buffer, and ready for PCR or stored at −20°C [11], [12].

### Real time-PCR for HR-HPV DNA

High-risk HPV (HR-HPV) infection was detected in an ABI Stepone thermal cycler with multiplex real time-PCR, the amplification was performed according to manufacture's protocol (Hybribio Biotechnology Limited Corp., Chaozhou, China).

The kits of real time PCR could detect 13 types of HR-HPVs infection (16,18,31, 33, 35, 39, 45, 51, 52, 56, 58, 59 and 68), but could not type.

### HPV GenoArray test

HPV GenoArray test was performed for HR-HPV positive samples with the same DNA as for real time PCR. Genotyping for HPV was done by DNA amplification, flow-through hybridization and gene chip by HybriMax (Chaozhou Hybribio Limited Corp., Chaozhou, China). Test was performed according to the manufacture's instructions. Detailed protocols for this assay had been described previously [12].The gene chip contained type-specific oligonucleotides immobilised on a nylon membrane. The chip could identify 13 high-risk HPVs (HR-HPVs) (16, 18, 31, 33, 35, 39, 45, 51, 52, 56, 58, 59 and 68), five low-risk HPVs (LR-HPVs) (6, 11, 42, 43, 44) and popular HPV type 53, 66 and CP8304 in Chinese population. The final results were detected by colorimetric change on the chip under direct visualization.

### Liquid-based cytology test (LCT) and pathological diagnosis

HPV DNA positive cases for real time-PCR were advised to receive LCT, which was performed in 3 hospitals (Chaozhou Central Hospital, Raoping People's Hospital and Chaozhou Gynecological and Pediatric Hospital). The women were called back and exfoliated cervical cells were collected, mixed with 3 mL specimen stored liquid (Hybribio, Chaozhou, China) and stored in room temperature. The specimens were sent to 3 above-mentioned hospitals for LCT analysis. The results were evaluated using the Bethesda system [13]. The evaluation system included: (i) negative, (ii) atypical squamous cells (ASC), (iii) low-grade squamous intraepithelial lesion, (iv) high-grade squamous intraepithelial lesion, and (v) squamous cell carcinoma.

The abnormal LCT cases were further advised to receive colposcopy and biopsies, all of cases with CIN 2, 3, invasive cancer or carcinoma in situ received loop excision, surgical conization, hysterectomy, or radiotherapy according to local protocols.

### Statistical analysis

All of data was analyzed by SPSS version 16 software. Chi-square test was used to assess HPV DNA infection rate in each age group and living area group. Linear regression test was used to assess the relationship between age and multiple HPV type infection constitute ratio. Statistical significance was accepted if the *P* value was 0.05 or less. All *P* values were two-sided.

## Results

### Prevalence of HR-HPV

At this program initiation, a total of 49458 women participated in the study, and finished the questionnaire, but 899 women did not meet the inclusion criteria and were excluded, mainly due to their age or total uterus or cervix resection (Figure 1).

Among 48559 eligible rural Chaozhou women, real time-PCR revealed that 3830 cases were positive for HR-HPV DNA, and the crude infection rate was 7.89% (3830/48559) (Figure 1).

**Figure 3:**
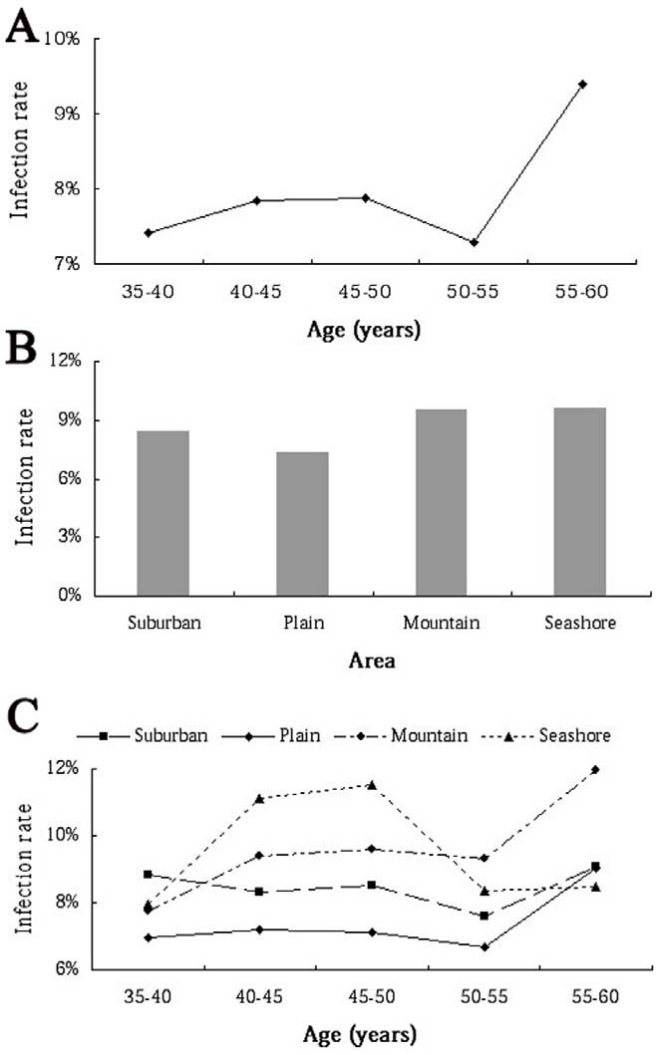
The HR-HPV prevalence. **A:** The prevalence of HR-HPV in 5 different age groups. **B:** The prevalence of HR-HPV in 4 different area groups. **C:** Specific age-related prevalence of HR-HPV infection in 4 different areas.

Age from 35 to 55, the prevalence of HR-HPV ranged from 7.28% to 7.88%, were all lower than the crude infection rates of 7.89%, there was no significant difference statistically between each group (P>0.05). However, the HR-HPV infection rate of age 55–60 group (9.39%) was significantly higher than those of the other 4 groups (P<0.001) (Table 1, Figure 3). Different from our previous report [12], the prevalence did not present a constantly increasing trend with age, instead of an infection peak at the oldest group (Figure 3A).

**Table 1:** Specific age-related prevalence for HR-HPV.

Age group	Total cases	Mean age (years)	HR-HPV positive cases	Prevalence
	no.	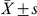	no.	%
**35–40**	9704	37.79±1.32	719	7.41
**40–45**	10920	42.55±1.45	856	7.84
**45–50**	10168	47.18±1.33	801	7.88
**50–55**	10151	52.55±1.39	739	7.28
**55–60**	7616	57.16±1.46	715	9.39*
**Total**	48559	46.95±6.78	3830	7.89

% = no. of HR-HPV positive cases/no. of total cases.

*The HR-HPV infection rate of women aged 55–60 group was significantly higher than those of the other 4 groups.

Women lived at the seashore had the highest prevalence of HR-HPV, the second was those lived in the mountain, the third was who lived in the suburban. The HPV infection rate in these three areas decreased from 9.61% to 8.45%, even though, it was still higher than the crude infection rates of 7.89%, and there was no significant difference between these 3 groups (P>0.05). Exclusively, the HPV prevalence of women lived in the plain (7.31%) was lower than the crude infection rate, and was significantly lower than the other three groups (P<0.01) (Table 2, Figure 3B).

**Table 2:** Specific area-related prevalence for HR-HPV DNA.

Area group	Total cases	Mean age (years)	HR-HPV positive cases	Prevalence
	no.	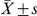	no.	%
**Suburban**	9170	47.35±6.92	775	8.45
**Plain**	31717	46.72±6.80	2320	7.31*
**Mountain**	6201	47.59±6.40	593	9.56
**Seashore**	1457	46.75±6.77	140	9.61
**Total**	48545	46.95±6.78	3828	7.89

% = no. of HR-HPV positive cases/no. of total cases.

*The HR-HPV infection rate of women lived in the plain was significantly lower than those of the other 3 groups.

Specific age-related prevalence was also observed in women who lived in the suburban, plain and mountain, there was an infection peak at the age from 55 to 60 years. However, there was a great different prevalence in women who lived at the seashore, herein, the HR-HPV infection rate increased in age from 35 to 50 years, and decreased in the elders (Figure 3C).

### Specific Prevalence of HR-HPV Types

3380 HR-HPV positive cases received HPV GenoArray test, 372 cases (11.01%, 372/3380) did not hybridize with any probes (21 subtypes), were coded as HPV type X. Among the typed 3008 cases, 101 cases were identified without HR-HPV type infection, and specific HR-HPV types were detected in 2907 cases, among them, 2209 cases (75.99%) were infected with a single HR-HPV type, and 698 cases (24.01%) were infected with multiple types (Figure 1).

Consistent with our previous report [12], type 52 was the most common HR-HPV type, more than one third cases (33.40%) were infected with this type (including simple infection and multiple infections). The other 5 most common HR-HPV types in descending order, were types 16 (20.95%), 58 (15.93%), 33 (9.94%), 68 (9.22%) and 18 (8.36%) (Table 3).

**Table 3:** The prevalence of 13 HR-HPV subtypes.

HPV type	Single infection		Multiple infections		Total	
	no.	%	no.	%	no.	%
**52**	654	22.50	317	10.90	971	33.40
**16**	397	13.66	212	7.29	609	20.95
**58**	310	10.66	153	5.26	463	15.93
**33**	153	5.26	136	4.68	289	9.94
**68**	146	5.02	122	4.20	268	9.22
**18**	150	5.16	93	3.20	243	8.36
**31**	98	3.37	92	3.16	190	6.54
**39**	115	3.96	58	2.00	173	5.95
**59**	55	1.89	41	1.41	96	3.30
**51**	44	1.51	25	0.86	69	2.37
**56**	39	1.34	24	0.83	63	2.17
**35**	26	0.89	21	0.72	47	1.62
**45**	22	0.76	24	0.83	46	1.58
**Total**	2209	75.99	688	24.01	2907	100

% = no./2907 (the total number of HR-HPV typed cases).

HR-HPV type was arranged by the descending order of infection rate.

**Figure 4:**
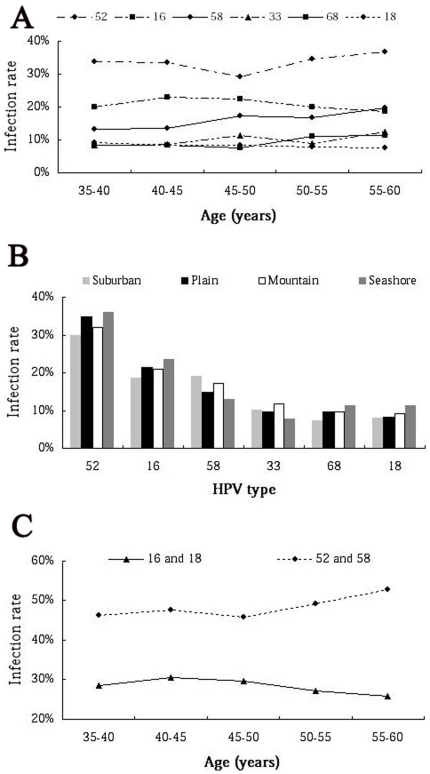
The prevalence of common infection HR-HPV subtypes in rural Chaozhou. **A:** The age-related prevalence of the 6 most common HR-HPV subtypes. For HPV subtypes 52, 16, 58, 33, 68 and 18, there was not apparent peak infection incidence in specific age group. **B:** The area-related prevalence of the 6 most common HR-HPV subtypes. For HPV subtypes 52, 16, 58, 33, 68 and 18, there was not any incidence difference statistically in women who lived in 4 different areas. **C:** The age-related combined prevalence of the type 16 plus 18, and type 52 plus 58.

In these 2907 HR-HPV typed cases, each HPV types presented a unique age-related prevalence, we could not find any incidence peak at the elder group corresponding to the prevalence of HR-HPV for real time PCR. Similarly, there was no lower incidence in women lived in the plain than those of the other three areas (Table 4 and 5). The age and area prevalence of the 6 most common HR-HPV types was shown in Figure 4 A and B.

**Table 4:** Specific age-related prevalence for each HR-HPV type.

HR-HPVType	Age 35–40		Age 40–45		Age 45–50		Age 50–55		Age 55–60	
	no.	%	no.	%	no.	%	no.	%	no.	%
**52**	191	33.75	222	33.38	180	29.27	182	34.47	196	36.77
**16**	113	19.96	152	22.86	138	22.44	106	20.08	100	18.76
**58**	75	13.25	89	13.38	106	17.24	88	16.67	105	19.70
**33**	48	8.48	58	8.72	70	11.38	47	8.90	66	12.38
**68**	48	8.48	55	8.27	46	7.48	58	10.98	61	11.44
**18**	52	9.19	56	8.42	52	8.46	42	7.95	41	7.69
**31**	40	7.07	50	7.52	43	6.99	27	5.11	30	5.63
**39**	41	7.24	45	6.77	27	4.39	30	5.68	30	5.63
**59**	16	2.83	25	3.76	22	3.58	15	2.84	17	3.19
**51**	13	2.30	15	2.26	15	2.44	13	2.46	13	2.44
**56**	10	1.77	14	2.11	10	1.63	17	3.22	12	2.25
**45**	10	1.77	12	1.80	9	1.46	9	1.70	6	1.13
**35**	14	2.47	11	1.65	5	0.81	10	1.89	7	1.31

% = no./the total number of HR-HPV typed cases in each age group.

Simple and multiple infections were all included in each age group.

**Table 5:** Specific area-related prevalence for each HR-HPV type.

HR-HPVType	Suburban		Plain		Mountain		Seashore	
	no.	%	no.	%	no.	%	no.	%
**52**	178	29.92	631	34.75	130	32.10	32	35.96
**16**	111	18.66	390	21.48	85	20.99	21	23.60
**58**	114	19.16	271	14.92	70	17.28	8	8.99
**33**	60	10.08	174	9.58	48	11.85	7	7.87
**68**	43	7.23	176	9.69	39	9.63	10	11.24
**18**	47	7.90	149	8.20	37	9.14	10	11.24
**31**	52	8.74	109	6.00	24	5.93	5	5.62
**39**	41	6.89	106	5.84	19	4.69	7	7.87
**59**	20	3.36	56	3.08	14	3.46	5	5.62
**51**	15	2.52	43	2.37	8	1.98	3	3.37
**56**	16	2.69	39	2.15	8	1.98	0	0.00
**45**	13	2.18	22	1.21	8	1.98	3	3.37
**35**	13	2.18	28	1.54	3	0.74	3	3.37

% = no./the total number of HR-HPV typed cases in each area group.

The combined prevalence of HPV types 16 and 18 accounted for 28.52% of total HR-HPV infected cases. However, type 52 plus 58 presented 48.23% of total HR-HPV infected cases. Interestingly, the prevalence of HPV type 16 and 18 decreased slowly with age increasing, while the type 52 and 58 increased with age increasing (Figure 4C).

**Figure 5:**
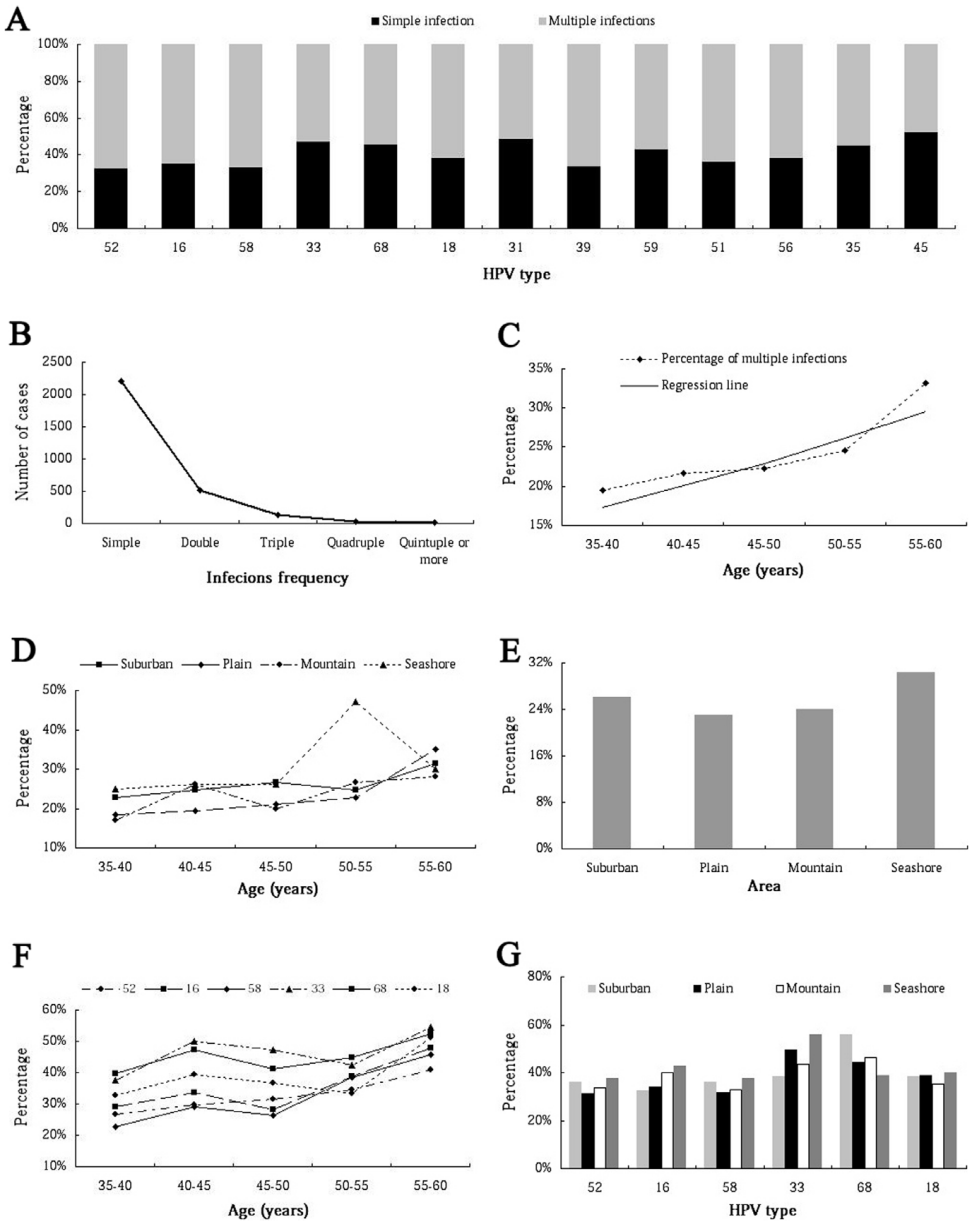
Simple/multiple infection composition. **A:** Simple/multiple infection constituent ratio of HR-HPV subtypes. **B:** The case numbers of infection frequency. The case number sharply decreased with the increasing of infection frequency. **C:** Linear regression relationship between multiple infections constituent ratio and age. The mean age of each group was 37.82±1.31, 42.47±1.47, 47.17±1.33, 52.60±1.41 and 57.13±1.43 years, respectively. The multiple infections constituent ratio increased with age. **D:** The age-related multiple infections constituent ratio in 4 different areas. The multiple infections constituent ratio increased with age for women who lived in the suburban, plain and mountain. For those lived at the seashore, there was an incidence peak in age from 50 to 55 group. **E:** The multiple infections constituent ratio in 4 different areas. There was no significant difference in 4 areas. **F:** The percentage of multiple infections of the 6 most common HR-HPV subtypes in 5 different age groups. **G:** The percentage of multiple infections of the 6 most common HR-HPV subtypes in 4 different area groups.

HR-HPV typically infected with a single type (Figure 5A), as described previously, more than three quarter (75.99%) genotyped cases were single infection. For multiple HPV infections, double infections was the major multiple infections, nearly three quarter (72.78%, 508/698) of multiple infection cases were double infections. About one fifth (139/698) cases were triple infections, and only 7% of cases were infected with four or more different HPV subtypes. At most, several cases were infected with seven different subtypes. The prevalence of infection frequency was shown in Figure 5B.

The most common infection type participated in the most common multiple infection state, for example, the type 52 combined with type 16 was the most common double infections. The main multiple HR-HPV infection types were shown in Table 6.

**Table 6:** Prevalence of HPV multiple infections subtypes.

Multiple HPV infection types	no.	%	Multiple HPV infection types	no.	%
**Double infections**	508	17.48	33 and 39	5	0.17
			33 and 18	4	0.14
52 and 16	42	1.44	33 and others^1^	19	0.65
52 and 58	19	0.65	68 and 18	3	0.10
52 and 33	19	0.65	68 and 39	2	0.07
52 and 68	18	0.62	68 and others^1^	15	0.52
52 and 31	18	0.62	18 and 31	5	0.17
52 and 18	16	0.55	18 and 39	2	0.07
52 and 39	7	0.24	18 and others^1^	15	0.52
52 and others^1^	72	2.48	Other double infections	50	1.72
16 and 58	21	0.72	**Triple infections**	139	4.78
16 and 18	12	0.41			
16 and 68	11	0.38	52, 68 and 31	6	0.21
16 and 33	9	0.31	52, 16 and 33	5	0.17
16 and 31	7	0.24	52, 16 and cp8304	5	0.17
16 and 39	5	0.17	52, 16 and 68	4	0.14
16 and others^1^	39	1.34	52, 16 and 58	3	0.10
58 and 68	10	0.34	52, 16 and 39	3	0.10
58 and 33	10	0.34	52, 18 and cp8304	3	0.10
58 and 31	5	0.17	52, 31 and cp8304	3	0.10
58 and 18	4	0.14	52, 58 and 53	3	0.10
58 and 39	3	0.10	52, 68 and 53	3	0.10
58 and others^1^	26	0.89	Other triple infections^2^	101	3.47
33 and 31	9	0.31	**Quadruple infections or more** 3	51	1.75
33 and 68	6	0.21			

% = no./2907 (the total number of HR-HPV infected cases).

1 Others included the lowest prevalence 5 HR-HPV type 59, 51, 56, 35 and 45, and LR-HPV type 6, 11, 42, 43 and 44, as well as Chinese popular HPV type 53, 66, and CP8304.

2 Other triple infections were less than 3 cases.

3 Two cases were infected with the same 4 subtypes (16, 18, 33 and 68), and two cases were infected with the same 5 subtypes (52, 56, 66, 68 and CP8304), the others were different from each other in that, there was only one case for every subtype combination.

Furthermore, we found out that the multiple infections constituent ratio increased with age, and there was a linear regression relationship, the Square R was 0.783, and the linear regression equation was: Y = −0.054+0.006X (Figure 5C). This kind of trend was also observed in women who lived in the suburban, plain and mountain. For women lived at the seashore, this kind of trend was found in age from 35 to 50, an incidence peak appeared in age from 50 to 55, and decreased in the elders. (Figure 5 D). But there was not significant difference for the multiple infections constituent ratio in 4 different areas (Figure 5E). Furthermore, this kind of age and area related prevalence was also observed in the 6 most common HR-HPV subtypes 52, 16, 58, 33, 68 and 18 (Figure 5F and 5G).

### Cytology test, diagnosis and treatment

A total of 2077 women infected with specific HR-HPV types received LCT. 12.3% (255/2077) women who had abnormal LCT received histological diagnosis, pathological abnormalities (CIN1 or above) were detected in 60.39% (154/255) women. A total of 67 cases with CIN3 or above were identified and they received appropriate treatment in hospitals. Among them, 26 (38.8%) cases were infected with HPV-16, 2 (3.0%) cases were infected with HPV-18, 2 (3.0%) cases were infected HPV-52, 3 (4.5%) cases were infected with HPV-58, the others (34 cases, 38.8%) were all multiple infection cases or infected with above not mentioned subtypes. Nearly 150 women infected with type X received LCT and 4 women were detected with pathological abnormalities (3 cases of CIN1 and 1 case of CIN2) and we did not detect CIN3 or above in type X group.

## Discussion

Until recently, a large-scale HPV testing for cervical cancer screening has been performed in China, based on the technology of Hybrid Capture II(HC- II) [14]. Real time multiplex PCR for HR-HPV test (Hybribio, China), which has already been used for quite a while in China, showed high level agreement with HC-II [15], [16]. With the support of local government, we organized a large-scale HPV testing for cervical cancer screening in rural Chaozhou based on the real time multiplex PCR. Considering that Real time multiplex PCR test could not identify genotypes, we utilized a GenoArray (Hybribio, China) for the detection of 21 HPV subtypes popular in Chinese, including 13 types of established HR-HPV.

Compared with similar studies in China, HR-HPV prevalence (7.8%) in rural Chaozhou of Guangdong Province was similar to that in Tibet (7.05%) [17], was also similar to Macao [18] and our previous study of urban women in Chaozhou (6.52%) [12], but lower than that in Beijing of north China (9.9%) [19], Zhejiang Province of southeast China [20], and an urban city in south China (17.6%) [21]. In Chaozhou rural area, about 300000 women between 35 and 60 years were eligible for this time screening. Although only about 20% of women participated, it may not cause large bias as HPV infection is asymptomatic and nearly 50000 (20%) samples was large enough [19]. The population in our study was asymptomatic women with no history of cervical neoplasia or other HPV-related diseases. Based on cost constraints, we did not perform the Bethesda System Terminology based cytology for all participants in this study, only HR-HPV women received cytology test.

The most common HR-HPV subtypes in general women population worldwide were HPV-16, -18, -31, -58, -52, -51 and -33, while the rank varied by area [22]. Contrasting to most previous studies in China [19], [21], [23], [24] and other populations [22], HPV-52, rather than HPV-16, was the most commonly identified type in our rural population (33.40% of all typed cases), consistent with the data from Guangzhou [25], Chaozhou city [12], Macao [18], Zhejiang province of China [20] and Japan [26]. HPV-16 ranked the second and the prevalence (20.95% of all typed cases) was comparable with that worldwide [22]. HPV-52 was more prevalent than HPV-16, this result may be due to the geographical and biological interplay between HPV types and host immunogenic factors [22], [27]. Another interpretation might attribute to the genotyping system [28], [29]. HPV-58, another common type in Asian population [18], [25], ranked the third (15.93% of all typed cases), similar to that in Japan [26]. An interesting point was HPV-68, not very common in other researches [3], [4], [22], ranked the sixth in our study, revealed the special distribution of HR-HPV in rural population of Chaozhou.

In our previous study, we found that HPV-52 and -58 were the most prevalent genotypes in Chinese women in Guangdong Province with HPV GenoArray-test kit [12]. Liu et al. reported that HPV GenoArray-test had a good sensitivity in detection of HPV-52, could also detect HPV-16 and -18 in as few as 10–50 copies [29]. The GenoArray-test and Roche Linear Array (LA) HPV genotyping assays showed no significant difference in the rates of detection of HPV genotypes, and the interassay agreement was excellent [29]. In addition, our subsequent sequencing studies suggested that GenoArray was a convincing method for genotyping (data not shown). Therefore, the preponderance of HPV-52 and -58, accounting for 48.23% of all infections in our study population, is very meaningful and convincing.

The screening included a round of real-time PCR detection for HR-HPV, and gene typing of a large sample of subjects for 13 subtypes of HR-HPV. However, there were 372 HPV DNA positive cases that could not be genotyped by GenoArray test (type X). We speculate that the most possible reason come from the difference between the sensitivity of real-time PCR and GenoArray test, the claimed sensitivity by manufacturer was 50 copies for the former, while 300 copies for the latter. Actually, we found that the mean cycle threshold (CT) value of these type X cases was much higher than the typed cases (data not shown). Therefore, we believed that the main reason for these type X cases was that they were infected with a relatively lower viral load.

For the current available prophylactic vaccines, the prevalence of vaccine types (HPV-16 and HPV-18) was consistently low across age groups in our study (28.52% of total typed cases). As our study had shown that HPV-52 and -58 were predominant in rural populations of Chaozhou, more prevalent than HPV-16 and -18, and we assume that the second-generation HPV vaccines including HPV-52 and -58 may provide higher protection for female in Chaozhou.

Bimodal age distribution of HR-HPV, which was common in previous studies [24], [25], was not observed in the current study. Older women (55–60 years old) in the Chaozhou rural area had the highest infection and the second was 50–55 years old group, suggesting a higher possibility of acquiring HPV infections at older ages [12].

As HPV is often acquired soon after sexual initiation [4], [5], the actual first peak of HR-HPV infection might before 20 years old, the age group not included in our research. Furthermore, older women (55–60 years old) in the Chaozhou rural area had the highest multiple infections and the multiple prevalence increased with age, there was linear regression relationship between multiple infections constituent ratio and age. The infection peak observed in older women of our study may be partly explained by viral persistence or reactivation of latent HPV due to the physiologic and immunologic disorder caused by hormone fluctuations at menopausal transition [12], [27]. HR-HPV positivity at the age of 55–60 years implies more possibility of viral persistence, which makes HPV screening more meaningful for older women than for younger women. For older female with positive HR-HPV, further evaluation, including cytology and even colposcopy, should be considered and regular screening should be prolonged because they have higher risk for the development of cervical cancer.

We also explored the effect of living area for HR-HPV infection, 4 kinds of typical living place were divided, including suburban, plain, seashore and mountain. There was significant difference between the incidence of plain and those of other 3 groups. suburban people lived neighborhood with city people, they were heavily influenced by city people, their incidence was also similar to the same city women [12]. In Chaozhou rural plain, because their well-developed industries, peoples here worked at their villages and seldom went out for work. However, at the seashore and mountain area, there were a lot of villagers work far away from their home (data not shown), that made husband and their wife separate, provide the opportunity for ex-marriage sex, that increase the infection possibility of HPV, because in areas where women's sexual conception and behavior are conservative, men possessing multiple sexual partners may be a predominant resource for HPV infection in female as long as HPV is mainly transmitted through sexual intercourse [30].

Previous studies showed that high-risk sexual behavior, including early sexual initiation, multiple sexual partners and sexual partner as an HPV carrier, was the vital factor for HPV infection in women [20], [31]. We also tried to explore the independent predictors for categories of HPV positivity, however, in rural Chaozhou, eastern Guangdong Province of China, a traditional Confucian culture influential place, most women refused to answer the question of sexual behavior, thus, we failed to get independent indicators related with sexual behavior that influenced the HPV infections.

HPV testing as a primary screening test for cervical cancer was supported by several reports [14], [26]. To our knowledge, this is the first population-based study conducted in rural Guangdong of China. This real time PCR testing for HPV-DNA (Hybribio) was rapid, simple, and affordable and its accuracy has been found to be substantially equivalent to the approach of using HC-II [15], [16]. In the context of successful establishment of a low-price, new real time HPV-DNA testing platform, using single HPV testing alone as the primary screening technology is promising in less developed areas where the health care infrastructure cannot afford a cytology-based screening system.

### Conclusions

We report the results of a population-based HPV screening of 48559 women in a rural area of Guangdong Province of China. The screening included a round of real-time PCR detection for HR-HPV, and gene typing of a large sample of subjects for 13 subtypes of HR-HPV. The population-based nature of this study provides previously un-available estimates of the prevalence of the full spectrum of HPV infections in this rural area of China. The cross-sectional information derived from this analysis, should aid in the design of HPV vaccines. The data showed low prevalence of available HPV vaccine types and relatively high prevalence of HPV-52 and -58. Our findings support the hypothesis that the second-generation HPV prophylactic vaccines including HPV-52 and -58 may offer higher protection for women in this rural area. Furthermore, our data also support close surveillance of 55–60 year women with HR-HPV infection. Our HPV-DNA screening trials could be easily performed and economically thrifty, will probably be conducted in other rural populations of China.
